# Health financing in Malawi: Evidence from National Health Accounts

**DOI:** 10.1186/1472-698X-10-27

**Published:** 2010-11-10

**Authors:** Eyob Zere, Oladapo Walker, Joses Kirigia, Felicitas Zawaira, Francis Magombo, Edward Kataika

**Affiliations:** 1 Inter-Country Support Team for East and Southern Africa, WHO Regional Office for Africa, Harare, Zimbabwe; 2 World Health Organization, Regional Office for Africa, Brazzaville, Congo; 3 WHO Country Office, Lilongwe, Malawi; 4 East, Central & Southern African Health Community, Arusha, Tanzania

## Abstract

**Background:**

National health accounts provide useful information to understand the functioning of a health financing system. This article attempts to present a profile of the health system financing in Malawi using data from NHA. It specifically attempts to document the health financing situation in the country and proposes recommendations relevant for developing a comprehensive health financing policy and strategic plan.

**Methods:**

Data from three rounds of national health accounts covering the Financial Years 1998/1999 to 2005/2006 was used to describe the flow of funds and their uses in the health system. Analysis was performed in line with the various NHA entities and health system financing functions.

**Results:**

The total health expenditure per capita increased from US$ 12 in 1998/1999 to US$25 in 2005/2006. In 2005/2006 public, external and private contributions to the total health expenditure were 21.6%, 60.7% and 18.2% respectively. The country had not met the Abuja of allocating at least 15% of national budget on health. The percentage of total health expenditure from households' direct out-of-pocket payments decreased from 26% in 1998/99 to 12.1% in 2005/2006.

**Conclusion:**

There is a need to increase government contribution to the total health expenditure to at least the levels of the Abuja Declaration of 15% of the national budget. In addition, the country urgently needs to develop and implement a prepaid health financing system within a comprehensive health financing policy and strategy with a view to assuring universal access to essential health services for all citizens.

## Background

The countries of the WHO African Region face critical constraints in financing their health systems to provide a basic package of cost-effective health care interventions deemed necessary to achieve the health-related Millennium Development Goals [1]. To improve the equity, efficiency and sustainability of their health financing mechanisms, many have undertaken health financing reforms as part of the broader health sector reform agenda [2].

The reforms that were launched in the 1980's and 90's were mainly focused on cost-recovery or cost-sharing schemes that aimed at collecting contributions from the users of public sector health facilities mainly through out-of-pocket payments or user fees [1]. However, the experience of user fees for health care in the region showed adverse effects in the form of, among other things, decreased utilization of services and impoverishment of households as a result of payment for health care.

Recognition of the negative consequences of out-of-pocket payments led to a growing consensus towards the need for prepayment schemes where people are required to make a regular contribution to health care through the payment of tax and/or insurance mechanisms. To this effect, the 58^th ^World Health Assembly passed a resolution in 2005 urging Member States to ensure that health financing systems include prepayment methods with a view to sharing risks and avoiding impoverishment of individuals as a result of seeking health care [3]. This was also reiterated by the WHO Regional Committee of Africa in its 56^th ^Session in 2006 [4].

The framework for implementing the Ouagadougou Declaration on Primary Health Care and Health Systems recommends that countries of the African Region develop comprehensive health system financing policies and strategic plans [5] in order to clearly chart the direction of their health financing systems towards achieving universal coverage with prepayment schemes. The development of health system financing policies and plans, however, requires a thorough analysis of the situation and a sound evidence base for meaningful intervention.

National Health Accounts (NHA) has been increasingly used in countries of the African Region to generate information and evidence on the state of health financing including its efficiency, equity and sustainability [6]. NHA is a framework for tracking and measuring total health expenditures and tries to address the following questions: (i) how are resources mobilized and managed for the health system? (ii) Who pays and how much is paid for health care? (iii) Who provides goods and services, and what resources do they use? (iv) How are health care funds distributed across the different services, interventions and activities that the health system produces? And (v) who benefits from health care expenditure [7]?

To date, many countries in the WHO African Region have conducted NHA at least once and utilized the findings to inform various policies and plans related to the sub-functions of health system financing that include the generation of resources; pooling and risk sharing; and resource allocation decisions [8].

This article attempts to present a profile of the health system financing in Malawi using data from NHA. It specifically attempts to document the health financing situation in the country and proposes recommendations relevant for developing a comprehensive health financing policy and strategic plan.

### Brief country profile

Malawi is a low-income country with an estimated gross national income (GNI) per capita of US$ 160 in 2004. Foreign aid, which amounted to US$37.8 million in 2004 constituted about 25% of the GDP [9]. The country reached the completion point under the Enhanced Heavily indebted Poor Countries (HIPC) Initiative and got approval of debt relief under the Multilateral Debt Relief Initiative in 2006 [10]. This implies that the country will save about US$ 110 million every year that was used to pay foreign debt [11].

In 2004/2005, about 52% of the population was classified as poor, i.e. below a national poverty line of Malawi Kwacha 16,165 per person per year - the equivalent of US$ 147. The incidence of poverty is higher in rural areas (56%) compared to urban (25%). It is also highest in the Southern Region of the country and female-headed households [12].

With a human development index (HDI) in 2005 of 0.437, the country is classified with the group of low human development countries, most of which are in sub-Saharan Africa. The country's HDI rank during the same period was 164 out of 177 countries. Trends in HDI indicate that, although there was a gradual increase in the HDI value from 0.3330 in 1975 to 0.444 in 1995, a decline was observed during the period 1995-2005 [13].

Malawi, like many countries in sub-Saharan Africa, faces a growing burden of disease and critical shortage of health system resources. The epidemiological profile is characterized by a high prevalence of communicable diseases including malaria, tuberculosis and HIV/AIDS; high incidence of maternal and child health problems; an increasing burden of non-communicable diseases and resurgence of the neglected tropical diseases. Although there has been a significant decline in infant and under-five mortality, the rates are still high. The maternal mortality ratio is one of the highest in the world. Access to health services is very limited. Some of the salient health indicators are presented in Table 1[14-16].

**Table 1:** Malawi socio-demographic indicators

Indicator	Value
Total population (2008)	13.1 million
Life expectancy at birth, 2007 (years) (both sexes)	50
Infant mortality rate, 2006 (per 1,000 live births)	69
Under-five mortality rate, 2006 (per 1,000 live births)	118
Maternal mortality ratio (per 100,000 live births)	984

The Government of Malawi is implementing a Health Sector Wide Approach (SWAp) since 2004. The delivery of the essential package (EHP) is central to the SWAp plan of work 2004-2010 [17]. The EHP, which comprises key health interventions against eleven diseases/conditions, is aimed at improving health outcomes through technical and allocative efficiency in service delivery; ensuring universal coverage of health services; and providing cost-effective interventions that address the priority health problems in Malawi. The cost of delivering the EHP was initially estimated to be US$ 17.5 per capita. However, later estimates have shown that the cost of delivering the EHP is much higher than the original estimate.

## Methods

The study is based on secondary data from three Malawi National Health Accounts (NHA) reports [18-20]. The first NHA exercise covered the Financial Year 1998/99; the second round included the Financial years 2002/2003, 2003/2004 and 2004/2005 and, in addition to the general NHA developed sub-accounts for three programme areas, namely HIV/AIDS, reproductive and child health programmes. The last one covered the Financial Year 2005/2006. Besides the general NHA, this also included sub-accounts for HIV/AIDS, malaria and tuberculosis. The focus of this study is on the general NHA. Implications of the NHA data are analysed in terms of their relevance to policy use within the framework of the various NHA entities.

### Definition of terms and core health financing indicators

In order to have a clear understanding of the essence of the report, it is important to present the definition of the most commonly used terms in NHA as provided by the NHA Producers' Guide [7].

i. Out-of-pocket payment: The direct outlays of households, including gratuities and payments in kind, made to health practitioners and suppliers of pharmaceuticals, therapeutic appliances and other goods and services whose primary intent is to contribute to the restoration or to the enhancement of the health status of individuals or population groups.

ii. Financing sources (FS): Institutions or entities that provide the funds used by the financing agents. They are the originators of the funds (e.g. Ministry of Finance, households, donors etc).

iii. Financing agents (HF): entities or institutions that channel funds provided from the financing sources and use those funds to pay for or purchase the activities inside the health accounts boundary. In the Malawian case, the financing agents included: Ministry of Health, National AIDS Commission, Other Ministries and Government Agencies (Ministries of Defense, Home Affairs, Education, Training Institutions, Regulatory Bodies-Nursing, Medical, Pharmacy and Poisonous Board etc), local authorities (Cities, Town and District Assemblies), private insurance enterprises (Medical Aid Society of Malawi), private households' out-of-pocket payment, non-governmental organizations (non-profit institutions), Christian Health Association of Malawi (CHAM), local non-governmental organizations, private firms and corporations, rest of the world (donors and international non-governmental organizations).

iv. Providers (HP): Entities that receive money in exchange for or in anticipation of producing the activities inside the health accounts boundary. They are the providers of health care services, e.g. hospitals, providers of ambulatory health care, pharmacies.

v. Health care functions (HC): The types of goods and services provided and activities performed within the health accounts boundary, e.g. curative care, services of rehabilitative care, prevention and public health services, health administration and health insurance, health-related functions (e.g. capital formation for health care provider institutions, education and Training of Health Personnel, research and development in health, environmental health and food, hygiene and drinking water control).

### Overview of the NHA methodology

The three Malawi National Health Accounts (NHA) studies used the standard internationally agreed NHA methods contained guidelines for low-income and middle income countries published by World Health Organization, World Bank and the United States Agency for International Development [7].

National health expenditure encompasses all expenditures for activities whose primary purpose is to restore, improve and maintain health for the nation and for individuals during a defined period of time [7]. National health accounts (NHA) are a tool for systematic, comprehensive, and consistent monitoring of resource flows in a country's health system. Specifically, the NHA tracks the flow of health system resources from financing sources (i.e. entities that provide the funds), financing agents (entities that receive and use funds to pay for health activities), providers (entities that receive money to produce health activities), functions (types of goods and services provided) and health system inputs to beneficiaries [7].

The total health expenditures consist of public funds, private funds and rest of the world funds. Public funds consist of mainly funds from central government revenue, regional and municipal government revenue and return on assets held by a public entity. The private funds compose of essentially employer funds, household funds and funds from non-profit institutions serving individuals. The rest of the world funds include bilateral grants, multilateral international grants and funds from funds contributed by institutions (including foundations) and individuals outside the country.

The commonly used national health accounts indicators include: levels of government and total per capita expenditure on health; total expenditure on health as a percentage of gross domestic product (GDP); general government expenditure on health as a percentage of total expenditure on health; private expenditure on health as a percentage of total expenditure on health; general government expenditure on health as a percentage of total government expenditure; external expenditure as a percentage of total expenditure on health; social security expenditure on health as a percentage of general government expenditure on health; out-of-pocket expenditure as a percentage of private expenditure on health; and private prepaid plans as a percentage of private expenditure on health. Sub-national health accounts entails producing those indicators by disease (e.g. HIV/AIDS, TB) or health programme (e.g. reproductive health, child health).

The three Malawian studies entailed populating with data the following four general (non-disease or program specific) NHA tables:

• Financing Sources (FS) × Financing Agents (HF);

• Financing Agents (HF) × Health Providers (HP);

• Financing Agents (HF) × Health Functions (HC); and

• Health Providers (HP) × Health Functions (HC).

However, the second and third studies in addition to the above mentioned general NHA tables they produced NHA sub-accounts of HIV/AIDS, Malaria and TB.

### Data Sources

The three studies used both primary and secondary data for financial years 1998/99, 2002/2003-2004/2005 and 2005/2006 collected from institutions and a specialist survey of People Living with HIV and AIDS.

The data was collected by trained research assistants, supervised by multi-sectoral NHA Technical Team of representatives from the relevant government ministries, United Nations agencies (WHO, UNDP and UNAIDS) and USAID-funded Partners for Health Reform*plus *Project (*PHRplus*). The co-authors EK and FM were closely involved in the design, analysis and writing of the three studies; and EZA was WHO's consultant in two of the studies. The questionnaires were tailored for each of the following data sources.

Firstly, data was collected from all the public sector institutions providing and receiving health funds, and providing health care goods and services, including: Ministry of Health, Ministry of Finance, Ministry of Foreign Affaires, Ministry of Local Government, Municipalities/Local Authorities, National AIDS Commission, Ministry of Defense and Home Affairs, Ministry of Education, Ministry of Women, Child Welfare and Community Services, Ministry of Agriculture, Nurses and Midwives Council, Medical Council; Pharmacy, Medicines and Poisonous board, and School of Health Sciences. The response rate was 100% in the three studies. The key informant interviews with those data sources was complemented with secondary data collection from government budget books, consolidated annual appropriation accounts, audited accounts, expenditure print-outs and ledgers.

Secondly, since no database exists for all donor (both bilateral and multilateral) expenditures on health in Malawi, a special donor survey targeting all 19 donors involved in health was undertaken to capture donor contributions for health using a specially designed questionnaire. The response rate for the first study was not noted. However, the response rates for other studies were: 40% and 47% in the second and third studies respectively.

Thirdly, in order to estimate NGO spending on health, a list of all NGOs and implementing agencies working in the health sector and HIV/AIDS sub-sector was obtained from Action Aid International Malawi. The list was reviewed to identify NGOs which were still functional during the time of the survey, in order to avoid sampling non-functional NGOs and community-based organizations (CBOs). Key informant interviews were used to select a total of 120 NGOs/CBOs. The response rate was 60% in the second and 47% in the third study.

Fourthly, data on employer and employees premium contributions to the medical aid society of Malawi (MASM), the only non-profit health insurance organization in the country, was obtained using a specially designed questionnaire. The quantity and quality of the data provided by MASM were excellent.

Fifthly, employers and employees in Malawi contribute to health expenditures through provision of onsite health facilities; reimbursements to employees; employer/employee contribution to an outside health insurance scheme, in particular MASM; and in-house health insurance scheme. A list of all firms and corporations registered in Malawi was obtained from the Malawi Chamber of Commerce. Key informant interviews were held and a comprehensive list of all firms involved in health and HIV and AIDS financing and delivery was prepared. Research assistants with a questionnaire were sent to all those firms. The response rates were: 80%, 65% and 86% in the first, second and third studies respectively. The information collected was supplemented by that collected from MASM.

Sixthly, surveys of purposely selected providers by different levels of care, ownership and region were carried out by research assistants and the NHA Team. The questionnaires were designed and used to collect the relevant information on utilization of various services, in particular for HIV/AIDS, reproductive health and child health (children age 0-5 years) and expenditure figures by source of finance/financing agent and function.

Seventhly, in order to estimate household out-of-pocket spending, national Integrated Household Survey results for 2004/05 were used. This estimated that health care consumes 1.3% of total private consumption. The figures were then distributed to various providers and functions using the household health expenditure and utilization survey of 2000.

Lastly, a People Living with HIV/AIDS (PLWHA) survey was conducted targeting confirmed HIV positive persons in Malawi age 15 years and older at the time of the survey. The major types of information obtained included utilization of health care services, household assets and expenditures for inpatient and outpatient care. Location sampling was used to identify the target population. The locations identified for the survey were: (a) PLWHA receiving ARVs in health centres and hospitals; and PLWHA receiving PMTCT. A sample of 900 individuals throughout the country was selected. The response rates were 93% in the second and third studies. The first study did not have sub-national health accounts.

### Data analysis

The data processing consisted of office editing of questionnaires, data cleaning (validation and consistency checks), data entry and analysis using Microsoft Excel software.

### Limitations of the three Malawi NHA studies

#### Reliability of estimates

data sources often provided conflicting data and lots of time had to be spent crosschecking and in some cases making value judgments of the data.

#### Incompleteness of data

Despite the research assistants' and NHA Team's repeated attempts at data collection, the response rate from donors and NGOs was poor and other sources had to be used to estimate their spending.

#### Unavailability of essential data in national health management information system (HMIS)

HMIS database did not have essential data on outpatient visits and inpatient admissions data by disease and facility type. Essential indicators such as bed occupancy rates, average length of stay, bed turnover rates, utilization by age, gender, type of facility-central hospital, district hospital, health centres are not reported to HMIS. Also it did not contain data by private-for-profit health sector.

#### Serious problems encountered with provider surveys

Funding and health services delivery are integrated at the health facility level making it extremely difficult for providers to disaggregate expenditures by source, function (curative, rehabilitative, ancillary services etc.) and disease type, e.g. HIV/AIDS. Furthermore, most private for-profit facilities were unwilling to provide their expenditures and revenue data, perhaps fearing that the data would be used for taxation purposes. Data on reported cause of morbidity or care seeking, number of bed days, discharge etc. were available in patient registers, but also were in a very poor state.

## Results

### Total health expenditure

In the Malawi National health accounts, total health expenditure is the sum of direct health care expenditures and capital investment. It should, however, be noted that depending on national policy purposes, countries may define total health expenditure differently. Some countries may, in addition to the above two items, include health-related activities such as health personnel training, health-related research and nutrition [7]. The trend in total health expenditure is presented in Table 2.

**Table 2:** Total and government health expenditure per capita, 1998/1999-2005/2006

Year	Total health expenditure (THE) per capita		THE per capita (US$) by source		
	
	US$	International dollars*	Public	External resources	private
1998/1999	12.4	40	3.2	3.6	5.6
2002/2003	15	50	5.3	6.9	2.8
2003/2004	17	66	3.8	10.6	2.6
2004/2005	20	70	5.1	12.0	2.9
2005/2006	25	85	5.4	15.2	4.6

* Local currency converted into dollar, adjusted for purchasing power parity

The total health expenditure per capita in 1998/1999 was about US$ 12 and increased steadily to US$25 in 2005/2006. This increase was achieved in nearly a decade. A breakdown of the 2005/2006 figure by sources of finance indicates that about US$ 15 per capita was contributed by donors, while government's contribution was only US$ 5 per capita.

### Sources of finance

The main financing sources in the 1998/1999 NHA were private sources contributing about 45% of the total health expenditure. However, in the later years, the major financing sources were donors as can be seen in Table 3.

**Table 3:** Source of health financing in Malawi, 1998/1999-2005/2006

Year	Percentage of total health expenditure			Out-of-pocket expenditure as percentage of private health expenditure
		
	Public	External sources	Private	
1998/1999	26	29	45	57.9
2002/2003	35.4	45.9	18.7	64.5
2003/2004	22.5	62.3	15.2	67.9
2004/2005	25.4	60	14.6	63.8
2005/2006	21.6	60.7	18.2	49.1

It can be observed from the above table that the share of private sources in the total health expenditure dropped by 27 percentage points, while that of external sources increased by 32 percentage points. The share of government exhibited a drop of about 4 percentage points during the stated period. Government contribution constituted less than a quarter of the total health expenditure in 2005/2006.

It is also observed that in the years up to 2005, about 2/3 of the contribution from private sources came from out-of-pocket payments. In the 2005/2006 Financial Year this has been reduced to about 50% implying a little increase in prepaid schemes. The out-of-pocket component of the private expenditure on health is still high even if a decrease of 14.7 percentage points was observed in the last year.

### Government expenditure on health

**Figure 1:**
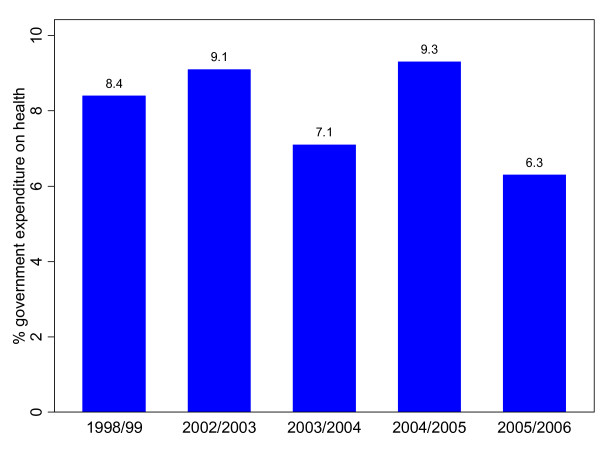
**Government expenditure on health as percentage of total government expenditure**.

Government expenditure on health was less than 10% of the total government expenditure over the period covered. This is far below the Abuja target, which committed governments in Africa to allocate at least 15% of their national budget on health [21] (Figure 1). If government allocation to the health sector as a proportion of total national government expenditure increased from its 2005/2006 level (6.3%) to the level of the Abuja Declaration (15%), government per capita expenditure on health would have increased to US$ 14.1. This increases the per capita total expenditure on health to about US$34.

There is no clearly discernible trend in government expenditure on health as a proportion of its total expenditure observed from the above figure. Government priorities seem to have been changing from one year to the other as indicated by the share allocated to health. It should, however, be noted that despite a decline in government allocation to health from 8.4% in 1998/99 to 6.3% in 2005/2006, there was a slight increase in absolute terms as seen in Table 2 above.

### Household expenditure on health

**Figure 2:**
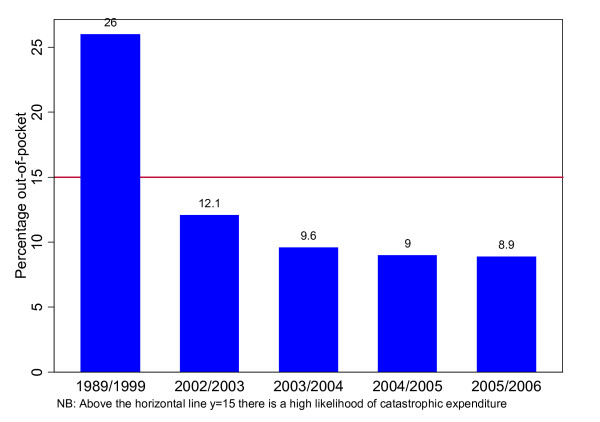
**Out-of-pocket expenditure on health as a percentage of total expenditure on health**.

The level of out-of-pocket payment in 1998/1999 was too high at 26% of the total expenditure on health (Figure 2). This is above the threshold for the incidence of catastrophic expenditure, which is set at about 15% [22]. Catastrophic expenditure is said to occur when households spend more than 40% of their disposable income after deducting subsistence allowances [22].

### Financing agents

Financing agents manage the funds received from financing sources. The distribution of total health expenditure by financing agents is presented in Table 4 for all the years except 1998/1999, which is not comparable to the subsequent NHAs in its design and structure.

**Table 4:** Percentage distribution of total health expenditure among financing agents, 2002/2003 - 2005/2006

Financing Agent	2002/2003	2003/2004	2004/2005	2005/2006
MOH	60.2	49.5	51.6	29.2
NAC*	1.8	3.5	11.9	4.4
other government agencies	0.7	0.8	0.7	4.4
local authorities	0.7	1.5	0.4	11.4
private insurance scheme	2.3	2	2.7	3.7
OOP**	12.1	9.6	9	8.9
CHAM***	4.2	2.9	4.2	3.2
Local NGOs	4.4	7.9	6.4	9.2
Private firms & employers	3	2.3	2.2	5.1
rest of the world	10.6	20	10	20.3

Note: * National Aids Commission; ** Out-of-pocket payment;

***Christian Health Association of Malawi

The role of the MoH as a financing agent was reduced in 2005/2006. In contrast, the role of local authorities and the rest of the world (donors) as financing agents increased remarkably. The share of the rest of the world as a financing agent doubled in 2003/2004 compared to that in 2002/2003. In 2005/2006, donors assumed the role of a financing agent almost equally as the Ministry of Health.

### Total health expenditure by health care providers

Providers are those entities that provide or deliver health care and health-related services. They address the NHA question "who provides health care services" or "where are services provided" [7]. Table 5 below presents the distribution of total health expenditure by health care providers.

**Table 5:** Distribution of total health expenditure by health care providers

Year	General hospital	Specialized hospital	Ambulatory health care	retail sale	prevention & public health	General health administration	Other
2002/2003	30.9	0.2	25.3	2	27.2	13.8	0.6
2003/2004	30	0.3	28.8	1.6	25.2	13	1.1
2004/2005	24.4	0.3	28.8	1.5	31.4	12.8	0.8
2005/2006	32.3	0.01	15.5	1.7	22.8	18.3	7.3

Note: 1998/99 has been excluded as the report does not contain this information

Although there was a decline in total health expenditure attributable to general hospitals between 2002/2003 and 2004/2005, a marked increase was observed in 2005/2006. Contrary to this, there was a marked reduction in ambulatory health care and prevention and public health programmes. General health administration showed an increase of about five percentage points. Again, there is no clearly discernible trend and that the situation in the last year seems to be inconsistent with the preceding years.

### Allocation of total health expenditure from financing agents to providers

The distribution of total health expenditure from financing agents to providers shows some irregularities, which may probably indicate problems in data availability and quality rather than substantive policy changes. For example, The MoH's (Financing agent) allocation to ambulatory care (provider) increased from 25% in 2002/2003 to 33.5% in 2004/2005 and decreased to less than 1% in 2005/2006 (Table 6). This is most unlikely to be true, as there is an atypical change in the last year under consideration and given the fact that providers of ambulatory care such as health centres and dispensaries/clinics play a significant role in service provision.

**Table 6:** Percentage allocation of total health expenditure from financing agents to providers

	Hospital				Ambulatory care				Retail sale				Prevention & Public health				Health administration & health insurance				Rest of the world				Provider not specified by kind			
	
	02/03	03/04	04/05	05/06	02/03	03/04	04/05	05/06	02/03	03/04	04/05	05/06	02/03	03/04	04/05	05/06	02/03	03/04	04/05	05/06	02/03	03/04	04/05	05/06	02/03	03/04	04/05	05/06
MoH	32.5	25	24.3	30.7	25	32	33.5	0.8	-	-	-	-	28.9	32	32	53	13.2	10.6	9.2	14.3	0.4	0.4	1	-	-	-	-	1.2
NAC	-	1	16.9	47	-	-	-	20.1	-	-	-	-	77.8	50.3	63.6	8.3	22.2	30.6	19.5	24.6	-	18.2	-	-	-	-	-	-
Other government agencies	-	10.7	-	-	18	-	14.4	5.4	-	-	-	-	-	59.1	58.7	36.6	82	30.2	26.8	21.9	-	-	-	-	-	-	-	36
Local authorities	-	-	-	57.2	8.6	30.8	12.9	42.4	-	-	-	-	84.4	29.6	87.1	0.4	7	39	-	-	-	-	-	-	-	-	-	-
Private insurance scheme	27.5	26.7	28.4	46	42.4	41.3	43.7	17.3	9.6	9.4	9.9	11.9	-	-	-	-	20	21.9	17.1	9	0.6	0.7	1	15.9	-	-	-	-
Household OOP	63.1	63.1	63.1	63.1	19.4	19.4	19.4	19.4	14.4	14.4	14.4	14.4	-	-	-	-	-	-	-	-	-	-	-	-	3.1	3.1	3.1	3.1
CHAM	51.4	46.3	53.6	48.6	16.4	19.5	22.8	23.9	-	-	-	-	16	16.8	14.5	16	16.2	17.3	9.1	11.4	-	-	-	-	-	-	-	-
Local NGOs	4.4	11.2	-	-	4.3	38.1	42.9	26.9	-	-	-	-	75	-	25.8	15.4	16.3	22.9	31.3	42.1	-	27.8	-	-	-	-	-	15.7
Private firms & employers	2.8	13.9	12.8	35.2	93.4	81.8	82.3	29.6	-	-	-	-	3.6	4.3	4.9	10.1	-	-	-	-	-	-	-	18	0.1	-	-	7
Rest of the World	7.7	43.8	10.7	23	28	21.1	26.8	10.4	-	-	-	-	36.8	21.4	38	13.7	27.5	13.7	24.6	36.9	-	-	-	-	-	-	-	16

Note: 02/03 represents the Financial Year 2002/2003; the same applies for the others

Allocation of household out-of-pocket payment to the various providers is seen to be constant over the years. The allocation of out-of-pocket health expenditure to the various providers is based on allocation factors obtained from household health expenditure and utilization survey of 2000. Thus there is a need for conducting another round of household health expenditure and utilization survey in order to obtain data that is useful for health financing policy purposes.

### Total health expenditure by health care functions

Distribution by health care functions shows preponderance to curative services, which consume nearly 50% of the total health expenditure (Table 7).

**Table 7:** Percentage distribution of total health expenditure by health care functions

Function	2002/2003	2003/2004	2004/2005	2005/2006
services of curative care	46.9	52.8	46.8	48.3
services of rehabilitative care	0.8	0.9	1.4	0.9
Medical goods dispensed to outpatients	2.0	1.6	1.6	1.7
Prevention and public health	27.3	25.2	31.4	22.8
Health administration and health insurance	13.8	13.0	12.4	18.3
capital formation	8.8	5.7	6.2	2.6
expenditure nsk*	0.4	0.9	0.3	4

* Not specified by kind

A decline was observed in the proportion of total health expenditure on prevention and public health, while there was an increase in the share of health administration and health insurance and services of curative care. However, the proportion of total health expenditure allocated to services of curative care is less than some countries in the region. For example, about 69% of the total health expenditure was allocated to services of curative care in Kenya in 2005/2006 [23]. Similarly, in the period 2001/2002-2006/2007, on average about 65% of the total health expenditure was allocated to curative care in Namibia [24].

### Discussion, conclusion and policy implications

This short report attempts to provide a **profile **of the health financing situation in Malawi using data from three rounds of NHA.

An increase was observed in the total health expenditure per capita over the years. However, it is still short of the recommendation of the Commission on Macroeconomics and Health, which recommended a spending of US$ 30-40 per capita to deliver a basic package of health services in low-income countries [25]. Moreover, after accounting for expenditure on health system administration and interventions not included in the Malawi Essential Health Package, the per capita total health expenditure of US$ 25 may not be able to finance the cost of delivering the essential health package, which at its design in 2002/2003 was estimated at US$ 17.5.

The share of private sources in the total health expenditure showed a declining trend. This decline is in the desirable direction. Apart from minimizing financial risks to households, this may also contribute to government and its partners' poverty reduction efforts in line with the Millennium Development Goals. Sustaining and intensifying this trend will require a continued support from external sources of finance.

The share of public funds in the total health expenditure manifested a decrease. This implies that there was an increasing dependency on donor resources. It is therefore important to develop strategies that will ensure the sustainability of the health system and the move towards universal coverage. Given the macro-economic reality of the country, donor support to the health sector will be necessary to maintain and increase the current levels of health spending and contribute to the positive changes in health status such as the remarkable drop in under-five mortality that the country has been able to achieve - with an average annual reduction rate of 6% in the period 2000-2008, the country is one of the few countries in Africa on track to achieve the MDG 4 target of reducing the under-five mortality rate by two-thirds between 1990 and 2015 [26].

The private component of the total health expenditure was dominant during the early years covered under the study accounting for nearly half of the total health spending. As the insurance sector is not well developed most of the contribution from private sources is attributable to household out-of-pocket payments. However, this has been reduced dramatically during the later years. This is a move in the right direction, as it contributes to reducing financial catastrophe and risk of impoverishment [27].

The Ministry of Health was the main financing agent managing more than half of the total health expenditure from 2002/3 to 2004/2005. However, its role was diminished significantly in 2005/2006. The share of the rest of the world (development partners) as a financing agent doubled in 2003/2004 compared to that in 2002/2003 as a result of procurement of anti-retroviral drugs by the United Nations Children's' Fund (UNICEF) using funds from the Global Fund to fight AIDS, tuberculosis and malaria [19]. The rest of the world managed health finances almost equally as the Ministry of Health in 2005/2006. This finding is partly explained by the fact that during this period donors such as the United States Agency for International Development (USAID) increased funding to vertical programs such as PEPFAR (the President's Emergency Plan for AIDS relief) [20]. Interestingly, this has been witnessed in the period after the country's health sector adopted the Sector-Wide Approach (SWAp) as a way of coordinating the activities of the various health development partners and during which a number of the major donors started pooling their resources and supported the Ministry of Health strategic and implementation plans. Such a situation may not be desirable as it may compromise the MoHs stewardship function. The increase in 2005/2006 of the role played by local authorities as financing agents **is **related to the implementation of the decentralization policy where health funds were transferred directly from the Ministry of Finance to the local authorities [20].

The distribution of the total expenditure on health by functions indicates preponderance to curative care. Moreover, an increase is observed in the proportion attributed to health system administration, while the proportion of prevention and public health has decreased. This may negatively affect the prevention and control of priority health problems including HIV/AIDS, malaria and tuberculosis and the upsurge of non-communicable diseases where health promotion and other preventive interventions play a vital role. The allocation of health system resources among the various health care functions, therefore, needs a closer look in order to achieve allocative efficiency.

The following policy implications are due from the foregoing discussion:

i. There is a need for increasing government's contribution to the total health expenditure to at least the levels of the Abuja Declaration of 15% of the national budget. This will increase the total health expenditure to levels that would cover the Malawi Essential health Package and possibly match the recommendations of the Commission on Macroeconomics and Health. However, to avoid potential problems related to absorptive capacity due to a relative increase in financing to the sector, appropriate measures need to be taken to strengthen performance of the health system.

ii. Given the economic situation of the country and current dependence on resources of the rest of the world, in the short to medium-term, there will be a need for a substantial contribution from development partners to finance health service provision. Therefore, it is necessary to develop a strategic relationship with partners for a sufficiently longer period and for predictability of funds so as not to jeopardize the sustainability of the health system and reverse the gains achieved in some of the MDGs such as the one related to reducing childhood mortality.

iii. In the face of increasing prevalence of non-communicable diseases and high burden of communicable diseases [28] that are amenable to primary prevention strategies and interventions, the decrease in the total health expenditure devoted to prevention and public health compromises allocative efficiency. It is therefore necessary to revisit resource allocation across various levels of national health system to ensure that more resources are invested in strengthening district health systems, including community-based outreach disease prevention and health promotion interventions.

iv. Prepayment schemes are still at a nascent stage. Hence, to facilitate the progress towards universal access to health care, it is necessary to develop and implement a comprehensive health financing policy and strategy as recommended in the 56^th ^WHO Regional Committee resolution [4] on health financing and the Ouagadougou Declaration [29].

v. Given the lack of consistency and questionable quality of data as demonstrated by some of the counter-intuitive and anomalous findings, it is necessary to invest in the development of a comprehensive health information system that collects health financing data on a regular basis and periodically undertake household health expenditure and utilization surveys. This will increase use of the NHA data to inform the development of appropriate health financing policies.

## Competing interests

The authors declare that they have no competing interests.

## Authors' contributions

EZ designed the study, performed the analysis and drafted the report; OW, JMK, FZ, FM and EK participated in the write-up and revision of the manuscript. All authors read and approved the final manuscript.

## Pre-publication history

The pre-publication history for this paper can be accessed here:

http://www.biomedcentral.com/1472-698X/10/27/prepub
